# Medical Items

**Published:** 1895-01

**Authors:** 


### *MEDICAL ITEMS.*

---

THE next meeting of the Missisippi Valley Medical Association will be held in Detroit.

---

DR. A. P. THORNTON, of Thornton, Tex., has been on a visit to relatives and friends in Atlanta.

---

THE Charity Hospital of Birmingham, Ala., was destroyed by fire December 1st. Loss about $40,000.

---

DR. F. L. SIM, of Memphis, died November 23d, in his sixtieth year. At the time of his death Dr. Sim was editor of the Memphis *Medical Monthly*, and Professor of the Practice of Medicine in the Memphis Medical College.

---

MISFORTUNES thick and fast seem to be falling upon our unesteemed quack-Doctor Flower, preacher, Christian scientist, etc., who used to make occasional flying visits to Georgia and diagnose diseases by intuition. He has recently been arrested both in Galveston and Chicago; in each case for "conducting a confidence scheme and obtaining money by false pretenses."

---

DOCTORS should be careful about buying preparations labelled "antitoxin." The temptation to manufacturing chemists is of course great, to offer this promising remedy for sale, but inasmuch as the process of manufacture requires several months, there is as yet no reliable antitoxin of American make. Thus far only the German or French preparation can be considered genuine or effective.

### THE DOCTOR'S WOOING.

BY ANGUS E. ORR.

'Tis said a very young M. D.
Once loved a maiden fair to see,
And thus this doctor, very young,
The lovely maiden's praises sung:

"How enchanting is thine azure iris
Shining 'gainst the white scleroid;
Gracefully attached the tendon
Of thy sterno-cleido-mastoid.

Capillary hyperæmia—
Of its tints the name is legion,
So pellucid is thy epidermis
In the oro-buccal region.

Of its glorious tints the sunset's
But a feeble imitator;
Oh, my darling, do but let me
Osculate your buccinator.

Than thy oral epithelium
Nought this side of Heaven is sweeter;
Graceful are the sinuous outlines
Of thy beautiful masseter.

Then thy zygomaticus major
Gives me joy almost divine
Whene'er it and thy risorius
In active league combine.

Empty is my pericardium,
Of its tenant thou bereft it;
Auricle and ventricle
And aorta's base have left it.

Yet I wish not to regain them;
No wish have I but that you
Will take also my peritoneum,
And my encephalon too.

Give me but thy sweet phalanges,
Thy metacarpals press to mine,
My cerebrum, cerebellum,
May they e'er be slave to thine.

Precious darling, come auscult me,
Thy concha 'gainst my thorax pressed.
On my blest manubrium ever
May thy precious cranium rest."

The doctor ne'er has ceased to wonder
Why she bade him go to thunder.

